# Effects of pathogen-specific clinical mastitis occurrence in the first 100 days of lactation 1 on future mastitis occurrence in Holstein dairy cows: An observational study

**DOI:** 10.3168/jdsc.2022-0354

**Published:** 2023-04-20

**Authors:** Julia A. Hertl, Ynte H. Schukken, Loren W. Tauer, Francis L. Welcome, Yrjö T. Gröhn

**Affiliations:** 1 Section of Epidemiology, Department of Population Medicine and Diagnostic Sciences, College of Veterinary Medicine, Cornell University, Ithaca, NY 14853; 2 Royal GD, 7418 EZ Deventer, the Netherlands; 3 Department of Animal Sciences, Wageningen University, 6700 AA, Wageningen, the Netherlands; 4 Charles H. Dyson School of Applied Economics and Management, Cornell SC Johnson College of Business, Cornell University, Ithaca, NY 14853; 5 Quality Milk Production Services, Department of Population Medicine and Diagnostic Sciences, College of Veterinary Medicine, Cornell University, Ithaca, NY 14853

## Abstract

The objective of this observational study was to estimate effects of clinical mastitis (CM) cases caused by different pathogens (*Streptococcus* spp., *Staphylococcus aureus*, *Staphylococcus* spp., *Escherichia coli*, *Klebsiella* spp., and CM cases with no growth) occurring in the first 100 d in lactation 1, of a dairy cow on the future rate of occurrence of different types of CM during a cow's full lifetime. The outcomes were occurrence of *Streptococcus* spp., *Staphylococcus aureus*, *Staphylococcus* spp., *Escherichia coli*, *Klebsiella* spp., and CM cases with no growth, after the first 100 d of lactation 1, until a cow's removal through death or sale in that or a subsequent lactation. Data, including information on CM cases, milk production, and event dates (including death or sale dates), were collected from 14,440 cows in 5 New York State Holstein herds from January 2004 until February 2014. Generalized linear mixed models with a Poisson distribution and log link function were fit for each pathogen. The individual cow was the unit of analysis. *Escherichia coli* was a predictor of future occurrence of *E. coli*, *Klebsiella* spp., and CM cases with no growth. Early-occurring *Klebsiella* spp. was a predictor of future cases of *Klebsiella* spp. Cases with no growth were predictors of future occurrence of *Staphylococcus* spp., *E. coli*, *Klebsiella* spp., and cases with no growth. Thus, *E. coli* and cases with no growth occurring early in lactation 1 appear to be consistent risk factors for future cases of CM, whether cases with the same pathogen or a different pathogen. In this study, farm effects on later pathogen occurrence differed somewhat, so treatment protocol and culling strategy may play a role in the findings. Nevertheless, the findings may help farmers in managing young cows with CM in early productive life, especially those with *E. coli* or cases with no growth, in that they may be more susceptible to future CM cases in their later productive life, thus meriting closer attention.

## Highlights

• Clinical mastitis occurrence in dairy cows over their lifetime was studied.
• Predictors were pathogen-specific CM cases occurring in the first 100 days of lactation 1.
• Outcomes were cases occurring after the first 100 days of lactation 1, until death or sale.
• *Escherichia coli* and cases with no growth were predictive of several CM types.
• The findings may help farmers predict which cows will have future CM cases.

## Graphical Abstract

**Figure:**
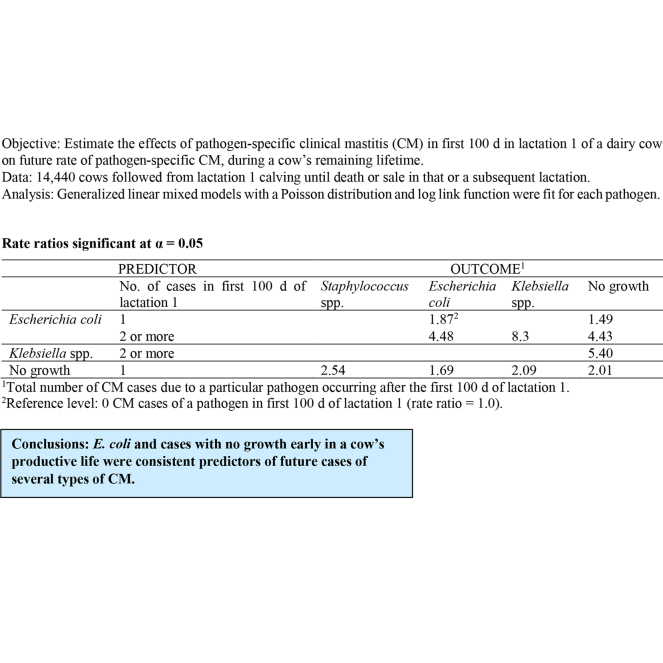
**Summary:** This study estimated the effects of pathogen-specific clinical mastitis (CM) occurring in the first 100 days of lactation 1 on the future occurrence of pathogen-specific CM in 14,440 Holstein cows in 5 New York herds. Cows were followed from lactation 1 calving date until death or sale. Generalized linear mixed models were fit. Clinical mastitis cases due to *Escherichia coli* or with no growth in the first 100 days were the most important predictors of future occurrence of pathogen-specific CM. The findings may help farmers foretell which cows that have CM in early productive life may have future cases.

Clinical mastitis (**CM**) is an important, costly disease in the dairy industry. It occurs throughout lactation, and a cow may have multiple occurrences, due to the same or different pathogens. Recurrent CM cases may occur as part of a cycle between subclinical and clinical episodes of the same (persistent) infection, or they may be different (re)infections following cure (Zadoks and Fitzpatrick, 2009).

It is of interest to know the importance of CM early in lactation 1. According to the International Dairy Federation (IDF, 2011), “early lactation” comprises the first 100 d of a cow's lactation. Persson Waller et al. (2021) reported that while mastitis is more common in older cows, a substantial proportion of lactation 1 cows also experience mastitis in the early stages of lactation. De Vliegher et al. (2012) reported that CM incidence in the first week of lactation was higher in lactation 1 cows than in older cows in New Zealand.

One study found that two-thirds of the CM cases in lactation 1 occurred in the first 30 DIM; CNS, *Streptococcus uberis*, and *Escherichia coli* were most frequently isolated (Barnouin and Chassagne, 2001). Schmenger and Krömker (2020) gave energy deficits due to onset of milk production and weaker immune system peripartum as a reason for their finding that most CM cases occurred in the first 100 DIM.

The risk of repeated mastitis cases in a lactation has been studied previously (Piepers et al., 2010; Cha et al., 2016), but rarely across lactations (Hultgren and Svensson, 2009). Moreover, the effect of CM occurring early in a cow's productive life on her future risk of contracting CM has only rarely been assessed recently (Santman-Berends et al., 2012; Hertl et al., 2018), but not at the pathogen-specific level. Cha et al. (2016) previously studied effects of a first CM case on the risk of a second case; and effects of a second case on risk of a third case, in the same lactation, for several different pathogens, but not specifically for the first case occurring early in a primiparous cow's life.

In a previous article (Hertl et al., 2018), we studied effects of CM occurring in early productive life, defined as the first 100 d of lactation 1, on the future rate of CM occurrence. In that study, CM cases were not differentiated by pathogen. For the current paper, we studied 6 pathogen groupings (occurring after the first 100 d of lactation 1) as outcomes: *Streptococcus* spp., *Staphylococcus aureus*, *Staphylococcus* spp., *Escherichia coli*, *Klebsiella* spp., and no important growth. These pathogen-specific cases of CM occurring in the first 100 d of lactation 1 served as risk factors for future cases of CM. Accordingly, the objective of this observational study was to estimate the effects of early-occurring (in the first 100 d of lactation 1) pathogen-specific CM on the future rate of CM (occurring after the first 100 d of lactation 1) due to the same or different pathogens.

The data set used in this study was a subset of the one used in Hertl et al. (2018). Data were available from 5 New York State herds from 2004 to 2014, serviced by Quality Milk Production Services (QMPS) of Cornell University, Ithaca, New York. All were conventionally managed and high-producing, and had fairly low SCC levels. Cows were housed in freestalls and managed in groups according to lactation month, production, and reproduction status. They were fed a balanced TMR and milked thrice daily. Data on calving, milk production, reproduction, and other events, including death or culling, were obtained from DairyComp305 herd management software (Valley Agricultural Software). These data were then cleaned, edited, and output to a form suitable for analysis.

The data set consisted of cows that had completed their productive lifetimes by the end of the data period [i.e., all had died or been culled (14,440 cows)]. All cows were followed from their lactation 1 calving date until death or sale in that or a subsequent lactation. This ensured having complete lifetime information on all cows.

Cases of CM occurred between January 2004 and February 2014. Milkers identified most cases during routine examination of fore-stripped milk before milking. A warm, swollen udder or change in milk consistency often occurred, indicating possible mastitis. Some cows had a sharp drop in milk yield (<70% of the average of the previous 10 d) or rise in milk conductivity (>115% of the average of the previous 10 d). Such cows were examined in further detail; if abnormal milk or a painful or swollen quarter was observed, CM was suspected.

Clinical mastitis cases exhibited the following signs: mild: abnormal milk (watery, flakes, fibrin clots, and so on); moderate: abnormal milk and swollen or painful quarter; severe: abnormal milk, swollen/painful quarter, and systemic signs of illness (fever, reduced appetite, dehydration, and so on). Milk samples from cows with CM signs were cultured at 1 of 3 QMPS laboratories in New York State (procedures detailed in Gröhn et al., 2004). The cows were assigned treatment and management protocols based on their clinical signs and causative pathogen; protocols were similar in all study farms.

If a second CM case occurred in the same udder quarter within 5 d after the first case (with either the same or a different pathogen), or occurred within 14 d after the first case (with the same pathogen), it was considered to be the same case. Any case occurring more than 14 d after the previous case, with either the same or a different pathogen, was deemed a new case (Barkema et al., 1998).

Generalized linear mixed models with a Poisson distribution (and log link function) (PROC GLIMMIX in SAS, version 9.4; SAS Institute Inc.) were fit for each pathogen. The individual cow was the unit of analysis. The outcome was the number of cases of a specific pathogen occurring after the first 100 d of lactation 1, with log(LifetimeDays) as the offset variable. Factors in the model were number of cases of each pathogen occurring in the first 100 d of lactation 1 [modeled as class variables (0, 1, and 2 or more cases)], farm [an indicator variable (1, 2, 3, 4, 5)], LifetimeDays (number of days from lactation 1 calving date until culling or death), LifetimeDays squared, and interaction terms between a set of binary indicators denoting whether or not the cow had any cases of a certain pathogen in the first 100 d of lactation 1 and LifetimeDays. These interactions adjusted the shape of the CM rate curve based on the lifetime of a cow having or not having CM in the first 100 d of lactation 1. For example, the model with *Streptococcus* spp. as the outcome looked like this (Equation 1):

[1] Log(*Streptococcus* spp. cases) = intercept + number of *Streptococcus* spp. CM cases in first 100 d of lactation 1 + number of *Staph. aureus* CM cases in first 100 d of lactation 1 + number of *Staphylococcus* spp. CM cases in first 100 d of lactation 1 + number of *E. coli* CM cases in first 100 d of lactation 1 + number of *Klebsiella* spp. CM cases in first 100 d of lactation 1 + number of CM cases with no growth in first 100 d of lactation 1 + LifetimeDays + LifetimeDays^2^ + LifetimeDays × [*Streptococcus* spp. in first 100 d (Y/N)] + LifetimeDays × [*Staph. aureus* in first 100 d (Y/N)] + … + LifetimeDays × [CM cases with no growth in first 100 d (Y/N)] + farm + log(LifetimeDays) [offset] + *e*,

where the outcome was the number of *Streptococcus* spp. CM cases in a cow's lifetime (excluding cases in the first 100 d of lactation 1), Y/N in the interaction terms for the pathogens indicates whether the cow had that pathogen in the first 100 d of lactation 1 (yes/no), and *e* denoted the error term. The offset variable, an adjustment term accounting for the fact that cows were followed for different lengths of time, was Log(LifetimeDays). An example of the data layout for several predictor variables with *Streptococcus* spp. as the outcome is given in Table 1. Exponentiating the model parameter estimates yields rate ratios (Table 2).

**Table 1:** Data layout for 7 example cows to study the effects of 6 types of pathogen-specific clinical mastitis (CM) occurring in the first 100 d of lactation 1 on the future rate of lifetime pathogen-specific CM (here, *Streptococcus* spp.), in 14,440 cows that died or were culled in 5 New York State farms, followed from January 2004 until February 2014^1^

Cow ID	Number of *Streptococcus* spp. CM cases in first 100 d of lactation 1	Number of *Staph. aureus* CM cases in first 100 d of lactation 1	Number of *E. coli* CM cases in first 100 d of lactation 1	Length of productive lifetime^2^ (d)	Total number of *Streptococcus* spp. CM cases in later lifetime^3^
1	0	0	0	1,367	0
2	1	0	0	808	0
3	1	1	0	1,523	0
4	0	0	2	799	1
5	2	0	0	458	5
6	1	1	1	574	3
7	1	0	1	956	1

1 Although only *Streptococcus* spp., *Staphylococcus aureus*, and *Escherichia coli* are listed here as example predictors, all 6 pathogen groups (*Streptococcus* spp., *Staphylococcus aureus*, *Staphylococcus* spp., *Escherichia coli*, *Klebsiella* spp., no important growth) were included as predictors in the models.

2 Productive lifetime: number of days from lactation 1 calving date until death or culling in that or a subsequent lactation.

3 Later lifetime: after first 100 d of lactation 1; values in this column comprise the outcome.

**Table 2:** Statistically significant rate ratios (i.e., different from a rate ratio of 1.0; including *P*-values), from generalized linear mixed models, for effects of number of cases of different pathogens in the first 100 d of lactation 1 on the rate of total number of clinical mastitis (CM) cases due to different pathogens in a cow's lifetime, in 14,440 cows in 5 New York herds followed from lactation 1 calving until culling or death in that or a subsequent lactation

Predictor		Outcome^1^					
Organism^2^	No. of cases in first 100 d of lactation 1^3^	*Streptococcus* spp.	*Staphylococcus aureus*	*Staphylococcus* spp.	*Escherichia coli*	*Klebsiella* spp.	No important growth
*Streptococcus* spp.	1	—4	—	—	—	—	—
	2 or more	—		—	—		—
*Staphylococcus aureus*	1	—	—	—	—	—	—
	2 or more			—	—		
*Staphylococcus* spp.	1	—	—	—	—	—	—
	2 or more	—	—	—	—		—
*Escherichia coli*	1	—	—	—	1.87 (*P* = 0.003)	—	1.49 (*P* = 0.049)
	2 or more	—	—	—	4.48 (*P* < 0.001)	8.30 (*P* = 0.0003)	4.43 (*P* < 0.0001)
*Klebsiella* spp.	1	—	—	—	—	—	—
	2 or more				—		5.40 (*P* = 0.04)
No important growth	1	—	—	2.54 (*P* = 0.003)	1.69 (*P* = 0.02)	2.09 (*P* = 0.04)	2.01 (*P* = 0.0002)
	2 or more	—		—	—	—	—

1 Total number of CM cases due to a particular pathogen occurring during full lifetime of a cow (after the first 100 d of lactation 1).

2 Total number of CM cases due to a particular pathogen occurring in first 100 d of lactation 1.

3 Reference level: 0 CM cases of a pathogen in first 100 d of lactation 1 (rate ratio = 1.0).

4 — = not significant at α = 0.05.

In the first 100 d of lactation 1, *Streptococcus* spp., *E. coli*, and cases with no growth showed the highest incidence rates (2.6%, 2.3%, and 2.8%, respectively). More than half of all cows had at least one case of CM in their lifetime. Over cows' entire lifetime, again, *Streptococcus* spp., *E. coli*, and cases with no growth occurred most often (22.8%, 22.5%, and 24.2%, respectively).

Farm-specific incidences ranged from 8.3% to 26.0% for *Streptococcus* spp., 2.0% to 15.0% for *Staph. aureus*, 1.5% to 15.9% for *Staphylococcus* spp., 10.8% to 21.9% for *E. coli*, 3.1% to 13.3% for *Klebsiella* spp., and 6.2% to 27.7% for cases with no growth, for all cases over lifetime during the study.

The rate of CM (due to any organism) increased slowly but steadily over productive lifetime, until 2,100 d, when it began to decline (Figure 1A). The sharp peak and drop after 2,500 d are likely caused by instability of estimates due to low case and cow numbers by this time. The rate of CM (due to any pathogen) over the first 100 d of productive lifetime is shown in Figure 1B. The rate was highest in the first 2 wk, then leveled off.

**Figure 1:**
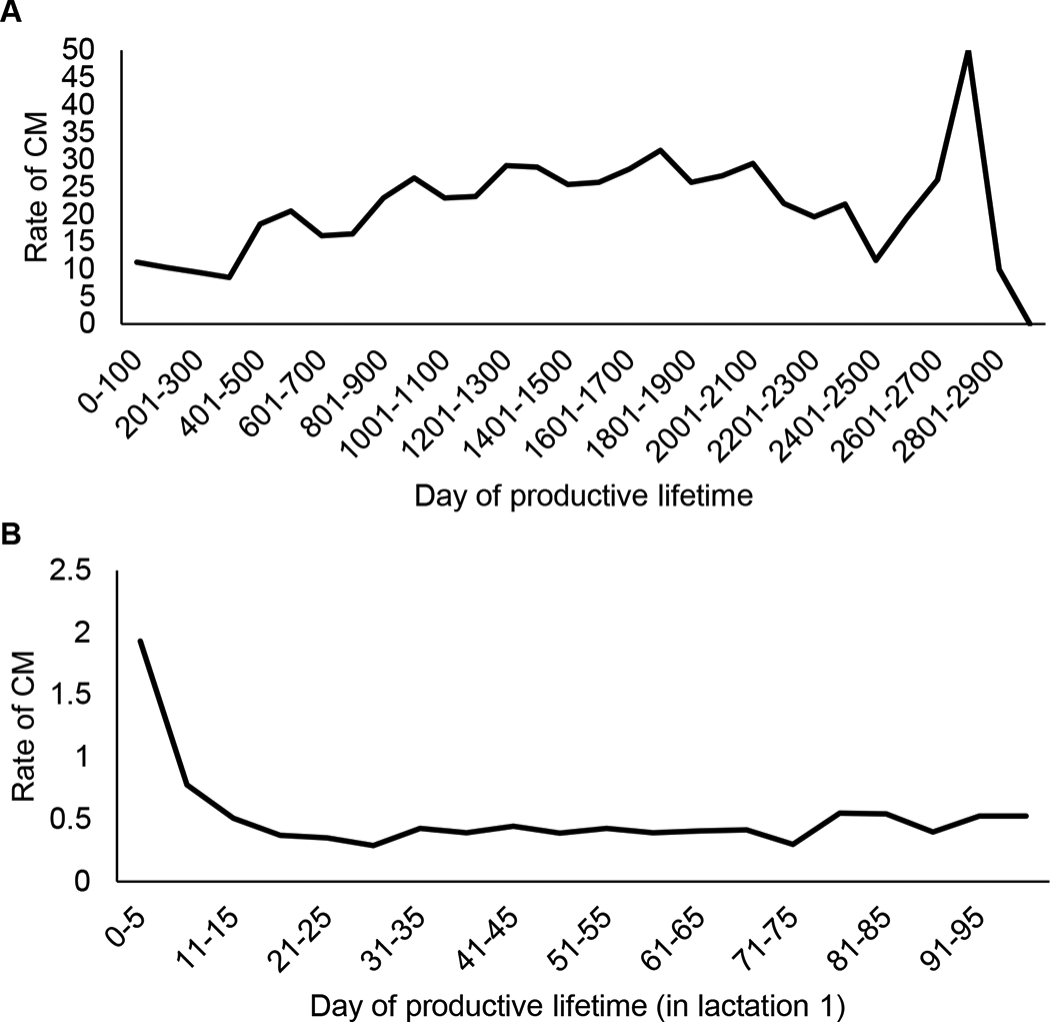
(A) Rate of clinical mastitis (CM; number of CM cases per 10,000 cow-days at risk) versus day of productive lifetime (beginning at lactation 1 calving date and ending at death or culling, including dry periods between lactations), and (B) rate of CM (due to any pathogen) in the first 100 d of lactation 1 (number of CM cases per 10,000 cow-days at risk) versus day of productive lifetime, in 14,440 cows in 5 New York State Holstein dairies, followed from 2004 to 2014.

Statistically significant rate ratios (at α = 0.05) are shown in Table 2. Early-occurring *E. coli* and CM cases with no important growth were consistently associated with occurrence of several other pathogens later in life.

None of the individual pathogens occurring early in productive life had a statistically significant impact on future occurrence of *Streptococcus* spp. or *Staph. aureus* CM.

Cows that had one case of CM with no growth in the first 100 d of lactation 1 had a 2.5 times higher rate (*P* = 0.003) of *Staphylococcus* spp. CM later on, than did cows with no cases of CM with no growth in the first 100 d.

*Escherichia coli* CM and CM cases with no growth occurring in the first 100 d of lactation 1 had an impact on future occurrence of *E. coli*, as did CM cases with no pathogen specified. Cows with one case of *E. coli* or with no growth in this early time period had nearly twice (*P* = 0.003; *P* = 0.02, respectively) the rate of *E. coli* later in productive life, whereas those with 2 *E. coli* cases early in productive life had a 4.5-fold higher rate (*P* < 0.001) of *E. coli* later on.

*Escherichia coli*, and cases with no growth, occurring in the first 100 d of productive life had impacts on future occurrence of *Klebsiella* spp. Cows with 2 *E. coli* cases in the first 100 d of lactation 1 had an 8.3 times higher rate (*P* = 0.0003) of *Klebsiella* spp. after the first 100 d of lactation 1. Cows with one CM case with no growth, in the first 100 d of lactation 1, had a 2-fold (*P* = 0.04) higher rate of *Klebsiella* spp. after the first 100 d of lactation 1.

Cows that had one, or 2 or more, *E. coli* cases in the first 100 d of productive life had 1.5- (*P* = 0.049) and 4.4-fold (*P* < 0.0001) higher rates, respectively, of cases with no growth after the first 100 d of productive life. Cows with 2 or more *Klebsiella* spp. cases, or one case with no growth, in the first 100 d of productive life had 5.4 times (*P* = 0.04) and 2 times (*P* = 0.0002) higher rates of CM with no growth after the first 100 d, respectively.

A few studies are available on recurrent cases of CM (and of the same pathogen; e.g., Döpfer et al., 1999; Cha et al., 2016; Wente et al., 2020), but none appear to focus specifically on CM cases occurring in the earliest stage of a cow's productive life as a risk factor affecting future CM cases in the cow's remaining productive life (i.e., over multiple lactations). The current study had 10 yr of data (i.e., multiple lactations) on CM cases and their etiology from 5 farms. With data on pathogen-specific CM cases during a cow's entire productive lifetime, our study was thus unique in these ways.

In the current study, only early-occurring CM cases due to *E. coli* and cases with no growth were consistent predictors of future CM cases due to various pathogens (Table 2). Zadoks and Fitzpatrick (2009) suggest that cases with no growth should actually be classified as being due to coliforms, where it was not possible to recover these organisms. Thus, these early cases may either result in long-term damage to the mammary gland or be an indicator of increased susceptibility. Cha et al. (2016) found that no protection was provided by previous CM episodes against subsequent ones, even when the same pathogens were involved. Schmenger and Krömker (2020) reported that CM cases due to *Strep. uberis* and coliforms had higher risks for recurrent infections than did NAS cases. Most of these reported cases were diagnosed in the first 100 DIM. They state as a possible reason that in early lactation, the immune system of cows is weaker due to negative energy balance at onset of milk production, leaving cows more vulnerable to infection. Zadoks et al. (2001) and Wente et al. (2020) also found higher rates of recurrence for *Strep. uberis*. *Escherichia coli* has also been associated with recurrent infections (Döpfer et al., 1999). These studies show that instead of providing protection against future infections, a CM case early in life is more likely to result in future cases, probably due to either persistent infections, increased genetic susceptibility, or long-term tissue damage.

The high rates of CM immediately postpartum in lactation 1 cows (Figure 1B) in this study are in agreement with various other studies (Barnouin and Chassagne, 2001; Piepers et al., 2010). This may be for several reasons, including poor hygiene, suckling (leading to infection spread), and so on. As well as these factors, primiparous cows are still growing, so are subject to further stresses on their physiology, in addition to all of the risk factors associated with first-time parturition and onset of milk production, which could leave them more vulnerable to infection compared with more mature cows. The CM incidence in lactation 1 cows in the first week of lactation in a New Zealand study was also higher than that in older cows; *Strep. uberis* and CNS were most commonly isolated from lactation 1 cows (De Vliegher et al., 2012).

Petersson-Wolfe et al. (2019) reported that up to 15% of lactation 1 cows are infected with *Staph. aureus* at calving, and can serve as a reservoir, and potentially spread through the herd. These infections may recur during the rest of lactation 1, and beyond (Petersson-Wolfe et al., 2019). This may explain the increased occurrence of *Staph. aureus* CM in older cows, although we did not observe this in the current study.

In a review of mastitis and biofilms, Gomes et al. (2016) stated that biofilms formed by some pathogens may play a role in recurrent infections because they inhibit therapeutic effectiveness and make them difficult to eradicate. They may also result in persistent infections (e.g., *Staph. aureus*; Zaatout et al., 2020). Mastitis pathogens that form biofilms in vitro include *Streptococcus* spp. and *E. coli* (Gomes et al., 2016), and *Staph. aureus* (Grunert et al., 2018).

Viruses may play a role in development of mammary lesions, facilitating occurrence of mastitis (Wellenberg et al., 2002). Teat injuries may also lead to opportunistic, pathogenic bacteria being able to establish mastitis infections. Such trauma may occur for many reasons, including incorrect milking or milk preparation procedures (Jones and Bailey, 2009).

A limitation of this study was that several species, namely *Strep. agalactiae*, *Strep. dysgalactiae*, *Strep. uberis*, and other *Streptococcus* spp., were grouped together into “*Streptococcus* spp.,” although differences may exist between them. It was not possible to perform further species-specific analyses, due to relatively low case numbers. This limitation also applied to *Staphylococcus* spp. Also, information on CM case severity was unavailable. With only 5 herds in this study, one must be careful regarding external validity. Farm-specific effects likely have some impact on CM occurrence and pathogen distribution. Although the general distribution of mastitis pathogens in this study is in line with observations in other studies (e.g., Barkema et al., 1998; Barnouin and Chassagne, 2001; Breen et al., 2009), it cannot be excluded that some specific farm or pathogen effects may be present. However, this would need to be reflected again in higher rates of future CM cases in cows that were observed with one or more CM cases in early life.

In conclusion, clinical cases of *E. coli* mastitis in the first 100 d of a cow's first lactation were predictive of future occurrence of *E. coli*, *Klebsiella* spp., and CM cases with no growth. *Klebsiella* spp. was predictive of cases with no growth. Clinical cases with no growth in the first 100 d were predictors of future instances of *Staphylococcus* spp., *E. coli*, *Klebsiella* spp., and cases with no growth. Thus, occurrences of certain types of CM early in a cow's productive life appear to be important risk factors for future cases of CM, whether with the same or a different pathogen. The knowledge gained in this study may be useful to farmers in managing young cows with CM in early lactation, especially those with *E. coli* or cases with no growth, in that they may be more susceptible to future CM cases, from various pathogens, in their productive life, thus meriting closer attention. Farms with a high incidence of CM with a given pathogen might want to control this pathogen particularly in young cows, as well as attempting to control the herd-level situation and reduce the incidence in all cows.
